# Single cell atlas of canine natural killer cells identifies distinct circulating and tissue resident gene profiles

**DOI:** 10.3389/fimmu.2025.1571085

**Published:** 2025-05-15

**Authors:** Aryana M. Razmara, Marshall Lammers, Sean J. Judge, William J. Murphy, Cameron E. Gaskill, William T.N. Culp, Alicia A. Gingrich, Zachary S. Morris, Robert B. Rebhun, C. Titus Brown, David M. Vail, Michael S. Kent, Robert J. Canter

**Affiliations:** ^1^ Division of Surgical Oncology, Department of Surgery, University of California Davis School of Medicine, Sacramento, CA, United States; ^2^ Department of Dermatology, University of California Davis School of Medicine, Sacramento, CA, United States; ^3^ Department of Surgical and Radiological Sciences, University of California Davis School of Veterinary Medicine, Davis, CA, United States; ^4^ Department of Human Oncology, University of Wisconsin School of Medicine and Public Health, Madison, WI, United States; ^5^ Department Population Health and Reproduction, University of California Davis School of Veterinary Medicine, Davis, CA, United States; ^6^ Department of Medical Sciences, University of Wisconsin School of Veterinary Medicine, Madison, WI, United States

**Keywords:** canine (dog), comparative, NK cell, single cell RNA sequencing (scRNAseq), transcriptomics, tissue resident

## Abstract

**Introduction:**

Natural killer (NK) cells in mice and humans are key effectors of the innate immune system with complex immunoregulatory functions, and diverse subsets have been identified with distinct characteristics and roles. Companion dogs with spontaneous cancer have been validated as models of human disease, including cancer immunology and immunotherapy, and greater understanding of NK cell heterogeneity in dogs can inform NK biology across species and optimize NK immunotherapy for both dogs and people.

**Methods:**

Here, we assessed canine NK cell populations by single-cell RNA sequencing (scRNAseq) across blood, lung, liver, spleen, and placenta with comparison to human NK cells from blood and the same tissues to better characterize the differential gene expression of canine and human NK cells regarding ontogeny, heterogeneity, patterns of activation, inhibition, and tissue residence.

**Results:**

Overall, we observed tissue-specific NK cell signatures consistent with immature NK cells in the placenta, mature and activated NK cells in the lung, and NK cells with a mixed activated and inhibited signature in the liver with significant cross-species homology.

**Discussion:**

Together, our results point to heterogeneous canine NK populations highly comparable to human NK cells, and we provide a comprehensive atlas of canine NK cells across organs which will inform future cross-species NK studies and further substantiate the spontaneous canine model to optimize NK immunotherapy across species.

## Introduction

Natural Killer (NK) cells are enigmatic innate lymphoid cells that uniquely blur the boundaries of innate and adaptive immunity due to their ability to recognize targets without prior sensitization while still possessing memory-like and tissue resident features (1–3). Equipped with an extensive array of activating and inhibitory receptors, NK cells use the dynamic engagements of these receptors to mediate effective antiviral and anticancer responses along with necessary tolerance to healthy cells and tissues.

Comparative oncology takes advantage of the similarities shared across species to identify and optimize treatments that are projected to have success in clinical trials (4–6). Although extensive studies have been completed in mice to advance NK immunotherapy, murine and human NK cells have significant differences in phenotype and function (intriguingly more than T cells) which have impaired successful clinical translation (7, 8). Of note, recent transcriptomic analyses have revealed greater homology between human and dog NK cells than human and mouse NK cells (9).

Numerous studies have also highlighted the heterogeneity of NK cells, including diverse subsets of NK cells across tissue compartments in both humans and mice. In humans, classical NK subsets include putative immature, CD56^bright^CD16^dim^ cytokine-secreting NK cells that predominate in lymph nodes versus mature cytotoxic CD56^dim^CD16^bright^ NK cell subsets that predominate in the blood (10, 11). Additional subsets include terminally differentiated NK cells found frequently in the lung (12) and mixed maturation state NK subpopulations in the liver (13). Importantly, identification of equivalent NK subsets in dogs has not been delineated. While spontaneous cancer in dogs presents a high incidence, high-fidelity model for translation to human cancers, studies are limited due to gaps in knowledge, especially for cancer immunology and immunotherapy, and these gaps extend to detailed understanding of NK subsets present in dogs. Additionally, it is paramount to address the differences between circulating and tissue resident NK cells to interpret tissue-specific responses in cancer, which can vary from those seen in the blood. Previously, circulating canine NK cells have been characterized using both flow-sorted CD3-NKp46+ and CD3-CD5dim subsets to characterize NK cell gene expression under multiple conditions, thereby identifying novel NK cell gene markers (9). Fortunately, high-quality canine reference genomes are available allowing for the use of single-cell RNA sequencing (scRNAseq) to investigate canine NK cells at the individual level with extensive markers for accurate identification and characterization (14).

To uncover the heterogeneity of canine NK cells throughout body compartments and identify potential targets for immunotherapy to optimize NK immunotherapy, we created a transcriptomic atlas of canine NK cells across blood and key tissues in dogs with a comparative analysis of human tissues for cross-species validation. We observed unique signatures across organs consistent with activated, differentiation-related, and conventional/regulatory NK cells in the lung, placenta, and liver, respectively. Importantly, we observed considerable overlap of canine tissue resident NK subsets with known subsets in humans. Together, our data inform the developmental trajectory and compartmentalization of NK cells in dogs and highlight potential strategies for improved translation of NK immunotherapy.

## Methods

### Sample acquisition and processing

Lung, liver, spleen, and placenta samples were obtained with owner consent from residual tissues of canine patients undergoing surgical procedures at the UC Davis Veterinary Medical Teaching Hospital. Donor characteristics for these dogs are listed in **Supplementary Table 1**. When applicable, only visually unaffected, non-tumor-bearing tissue was used. Processing of spleen and placenta consisted of mechanical digestion followed by incubation with RBC lysis buffer for five minutes at 4°C. Liver processing included mechanical digestion followed by a cell separation step using Percoll and PBS+3% FBS before RBC lysis (15). Lung processing included mechanical digestion followed by enzymatic digestion using DNAse and collagenase (16). PBMCs were isolated from whole blood from healthy beagle donors (Ridglan Farms). Processing was performed as described previously (9, 17, 18).

For human samples, the collection of blood and residual tissue specimens was obtained after informed patient consent per the IRB at the University of California, Davis (Protocol #218204 UC Davis Pathology Biorepository - Tissue, Blood, Urine and Other Biological Material). Human samples were processed as described previously (9, 17, 18). Donor characteristics for these patients are listed in **Supplementary Table 2**.

### Fluorescence-activated cell sorting

After processing, normal dog lung, liver, and placenta cells were washed with PBS and staining buffer. Canine cells were incubated with Fc receptor blocking solution (Canine Fc Receptor Binding Inhibitor, Invitrogen #14-9162-42), and stained with rat anti-dog monoclonal antibody CD45-EF450 (clone YKIX716.13, Invitrogen #48-5450-42). Human cells were incubated with Human TruStain Fc receptor blocking solution (BioLegend, #422302) and stained with mouse anti-human monoclonal antibody CD45-BV510 (clone HI30, BioLegend). Live/dead discrimination was performed using Fixable Viability Dye 780. Cell sorting for live CD45+ cells was performed using the Becton Dickinson “Aria II” Cell Sorter (Becton Dickinson, San Jose, California, USA).

### Single-cell RNA sequencing and analysis

Single-cell suspensions of 700–1200 cells/µL with a minimum of 40 μL of PBS/0.5% bovine serum albumin suspension buffer were submitted for library preparation and sequencing using the 10X Chromium Next GEM Single-Cell 3’ V.3.1 Gene Expression protocol performed by the UC Davis Genome Center as previously described (17). Human (GRCh38) and canine (CanFam3.1) indexes were created using the *cellranger mkgtf* and *cellranger mkref* pipelines. Raw fastq files were aligned to the relevant reference genome and feature-barcode matrices were created using CellRanger v.7.1.0 (10x Genomics). The feature-barcode matrices were uploaded in Rstudio and analyzed using Seurat. Seurat objects were created with a minimum cell threshold of 3 and minimum features of 200. Only cells with ≤15% of mitochondrial counts and unique feature counts ≥200 and ≤5,000-6,000 were filtered for analysis. Data then underwent standard Seurat processing workflow which included normalization, identification of highly variable features (2,000), and scaling. Cells were then clustered through a standard workflow that included linear dimensional reduction, determination of the k-nearest neighbor (KNN) using the top PCs based on the generation and interpretation of an elbow plot, and then implementation using a resolution of 0.5 after testing of multiple PCs and resolutions. Doublets were then identified and removed using DoubletFinder before the cell clustering workflow was repeated with doublets and unwanted cells removed.

For merged analyses, samples were integrated using Harmony (19). Layers were joined and cells clustered using 50 PCs and resolution of 2. For subset datasets, cells identified as NK cells were subset followed by normalization, identification of variable features, scaling, PCA, clustering and generation of UMAP. Differential gene expression testing was performed using the FindAllMarkers function in the Seruat R package to identify differences between two identified groups using the Wilcoxon Rank Sum test. Genes were only considered significant if the adjusted p-value, using Bonferroni correction, was p<0.05.

Tissue signatures were developed by determining significantly different genes between NK cells within one tissue compared to NK cells in all remaining tissues. The list of DEGs was then filtered to include only genes that had an adjusted p-value <0.05, average log fold change >1.0, and had expression in at least 20% of NK cells. Representative genes were selected and categorized by associations obtained from public databases (EnrichR, Uniprot, and NCBI).

To infer cell-cell interactions across all tissues as well as within individual tissues, CellChat was used (20). The strengths and weights of interactions were based on the CellChat ligand-receptor interaction database. Pseudotime analysis was completed using Monocle3 with clustering using a resolution parameter set to 1x10–^3^ and the root set based on visualizations of cluster IDs, partition assignments, and tissue of origin.

## Results

### Canine NK cells vary in abundance and immune interactions across tissues

**Figure 1:**
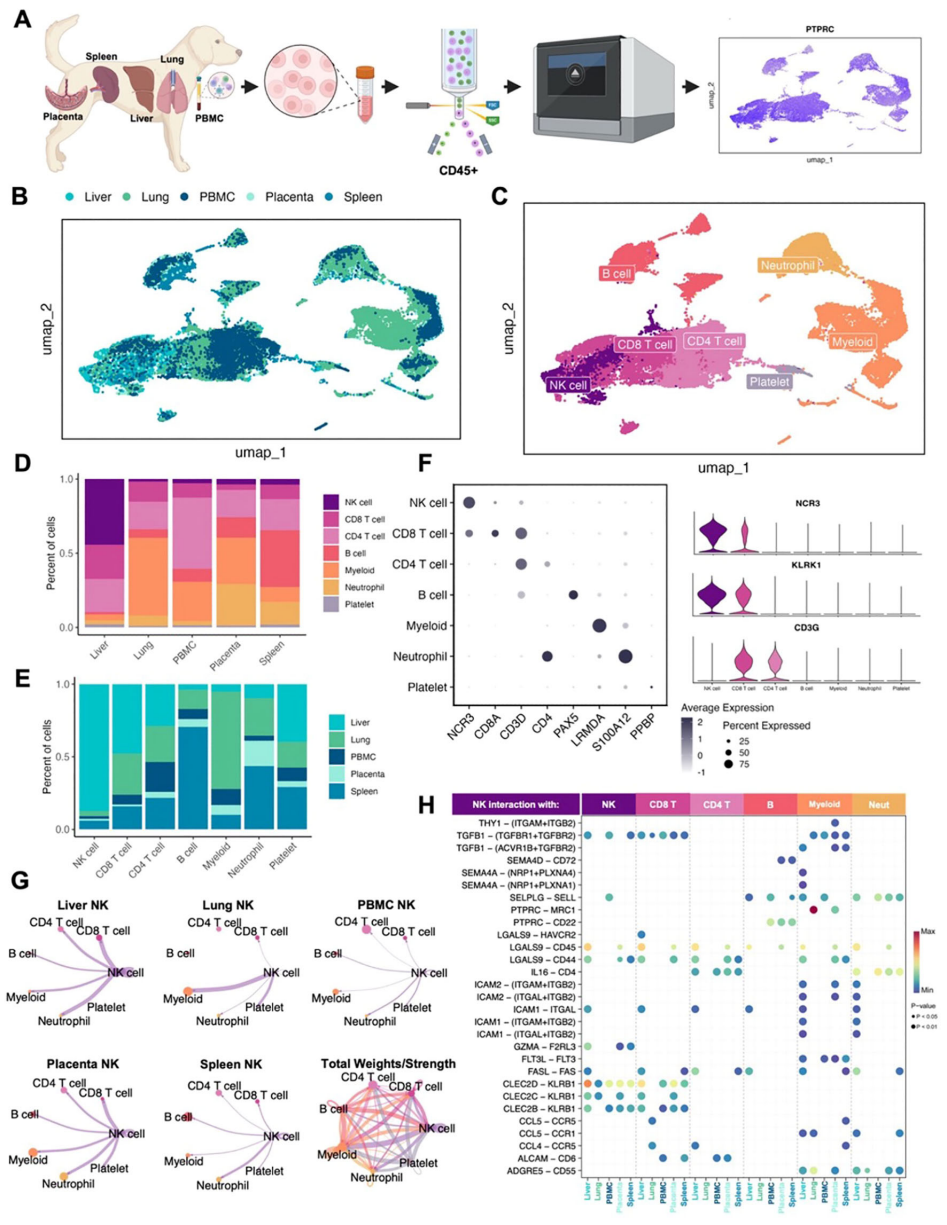
Canine NK cells vary in abundance and immune interactions across tissues. **(A)** Schema depicting the canine samples obtained and study workflow. **(B, C)** Uniform manifold approximation and projection (UMAP) visualizations of integrated samples encompassing nine total samples color coded by **(B)** tissue or **(C)** cell type. **(D, E)** Bar plots depicting the percent of cells analyzed by **(D)** tissue compartment or **(E)** cell type. **(F)** Left: dotplot of representative canonical gene markers used to confirm cell types. Dot color represents average gene expression and dot size represents the percent of cells expressing the gene. Right: violin plot of key genes distinguishing NK cells from T lymphocytes. **(G)** Circle plots constructed using CellChat visualizing predicted NK cell outgoing interactions separated by tissue. Lines represent the scaled weights or strength of the interaction. Total weights and strengths of ingoing and outgoing interactions between all cell types across tissues visualized in the bottom right panel. **(H)** Bubbleplot of significant predicted ligand receptor interactions between NK cells and other cell types, separated by tissue. Color of dot represents the probability of the interaction, and size of dot corresponds to p-value. Only interactions expressed in a minimum of 10 cells were retained in the analysis.

To understand canine NK cell heterogeneity, activation, and maturation states, we created a transcriptomic atlas of canine NK cells across tissues and peripheral blood. We used two samples each of canine placenta, spleen, liver, and lung from different donors in addition to a single blood sample from a healthy beagle donor.CD45+ cells were flow sorted to enrich for immune cells prior to performing scRNAseq (**Figure 1A**; **Supplementary Figure S1A**). After quality control and processing, samples were integrated for a dataset of approximately 50,000 high-quality cells available for analysis. We used Harmony integration to remove batch effects and allow for cell grouping based on cell type rather than donor or tissue (19), as demonstrated in uniform manifold approximant and projection (UMAP) plots color coded by tissue or cell type (**Figures 1B, C**). Cells were annotated manually as well as by using the AddModuleScore function based on markers and gene lists from relevant literature. Immune populations, including NK cells, varied across tissues (**Figures 1C, D**) with the largest proportion of NK cells found in the liver, making up nearly 45% of CD45+ cells present. Proportions of other cell types also matched known cell distributions in tissues including large populations of myeloid cells in the lung and B cells in the spleen (**Figures 1D, E**). Immune cell identities were determined by interrogation of cluster-specific gene markers against canonical immune cell type markers. Canine NK cells were confirmed as expressing NCR3 and KLRK1 but lacking the CD3 expression seen in CD4 and CD8 T cells (**Figure 1F**), previously identified as a transcriptomic signature of canine NK cells in the blood (21). Since cell-to-cell communications and cues vary based on maturation and activation states, we used CellChat to further predict cell interactions within tissues (20). NK cells in the lung had the strongest interactions with myeloid cells, which is particularly relevant due to the immunoregulatory interactions between NK and myeloid cells in the tumor microenvironments (**Figure 1G**). Meanwhile, NK cells in the spleen had minimal interaction with myeloid cells and stronger interactions with neutrophils. The combined tissue circle plot depicts a complex network of cell interactions across all cell types (**Figure 1G**). These interactions were further elucidated by determination of all significant ligand receptor interactions between NK cells and other cell types (**Figure 1H**). The SELL-SELPLG interaction between NK cells and other NK cells, B cells, neutrophils and myeloid cells was previously appreciated in treatment naive canine osteosarcoma between NK cells and mature regulatory dendritic cells (22). The two interactions with the highest probability were PTPRC-MRC1 in myeloid cells in the lung and CLEC2D-KLRB1 with other NK cells in the liver (**Figure 1H**). The CLEC2D-KLRB1 interaction has been noted between myeloid and NK cells in human neuroblastoma and between tumor and CD8 T cells in human rhabdomyosarcoma (23, 24), pointing to the presence of immunosuppressive networks in the liver.

### Canine NK cells demonstrate tissue-specific gene signatures

**Figure 2:**
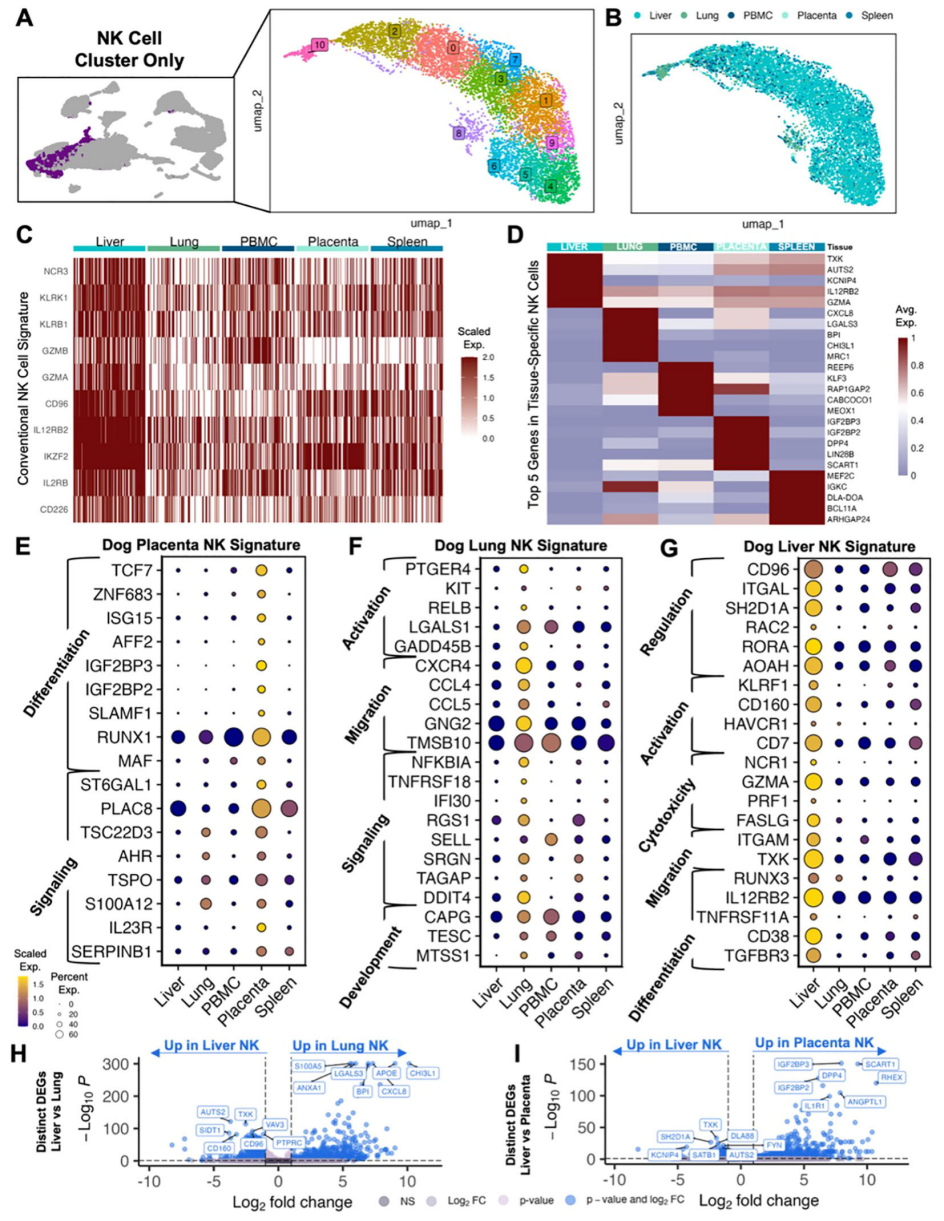
Canine NK cells demonstrate tissue-specific gene signatures. **(A)** UMAP representation of NK cells subset from the integrated dataset with 11 clusters identified by unsupervised clustering. **(B)** UMAP visualization of NK cells color coded by tissue. **(C)** Heatmap of conventional NK cell markers in NK cells analyzed by tissues. Columns represent cells and rows represent genes. Gene expression data for all features/genes were scaled and a random downsample of 100 NK cells from each tissue were plotted for proportional visualization. **(D)** Heatmap showing the average expression of the top five differentially expressed genes in NK cells from each tissue that distinguish them from NK cells in all other tissues. **(E–G)** Dotplots showing expression of representative genes significantly upregulated in **(E)** placenta, **(F)** lung, and **(G)** liver NK cells compared to NK cells in all other tissues. Genes included were present in >20% of cells, with average log2FC>1 and adjusted p-value<0.05. Gene category labels were determined by gene-set library and gene ontology annotations associated with each gene. **(H, I)** Volcano plots with labels for the top seven significant genes that differentiate **(H)** liver vs lung NK cells and **(I)** liver vs placenta NK cells based on direct DEG comparison.

To better understand the characteristics of canine NK cells and their diversity across tissues, we then analyzed the NK cell clusters from the integrated dataset and performed unsupervised clustering for higher resolution analysis of these cells. The majority of these cells were from liver (n=6,643) (**Figures 2A, B**). NK cells across tissues expressed genes associated with a conventional NK cell signature, including NCR3, KLRK1, and GZMA (**Figure 2C**). The topmost significant genes of tissue-specific NK cells when compared to all remaining NK cells were interrogated to elucidate the heterogeneity of NK gene expression (**Figure 2D**). Liver NK cells had increased expression of conventional NK genes including IL12RB2 and GZMA, the latter being identified as one of seven genes that drive human NK cell subpopulations (25). Additionally, NK cells in the lung expressed CXCL8, which is induced in activated NK cells leading to the migration and activation of other cell types such as dendritic cells, as well as CCL4 and CCL5 which have been used to characterize the functional capacity of NK cells in human lung (26, 27). We then analyzed these significant gene sets to determine signatures that were representative of individual tissues. In the placenta, we identified differential gene expression for genes associated with differentiation and signaling (**Figure 2E**). The involvement of RUNX1 and TCF7 in NK cell maturation has been well-documented (13, 28, 29), and TCF7 expression has been inversely correlated with lymphocyte exhaustion in human breast cancer (30). Genes associated with activation and migration were significantly enriched in lung NK cells (**Figure 2F**). Of note, we observed upregulation of multiple immune recruitment associated genes, such as CXCR4, CCL4, and CCL5, implicating lung NK cells in the orchestration of coordinated multi-cellular immune responses. Notably, the expression of these genes, particularly CCL4, has been associated with the activated and mature, effector CD56^dim^ classification in human NK cells (10, 25, 28, 31–33). Interestingly, NK cells in the liver expressed mixed gene signatures with genes associated with immunoregulation differential expression in combination with genes associated with activation and differentiation (**Figure 2G**). In the liver, one of the most recognized inhibitory receptors in NK cells, CD96, was upregulated simultaneously with cytotoxicity receptors GZMA and FASLG, indicative of the sensitive balance of opposing receptors that regulate NK responses. We also observed expression of the activation marker, CD160, known to be enriched in NK cells identified in hepatocellular carcinoma (32), but also potentially a marker of early, tissue-resident ILC1 cells (13). It is possible that our NK cell populations include ILC contamination and additional work is needed differentiate this critical population since ILCs have not been thoroughly characterized in the dog. Like the placenta, the liver also had increased expression of RUNX3 and IL12RB2, both associated with NK precursors and differentiation (34, 35). Due to the overlapping characterization of liver NK cells with both placenta and lung NK cells, we then determined the key differences between these tissue-resident NK cells with direct comparisons of liver and lung NK cells and liver and placenta NK cells (**Figures 2H, I**). In both lung and placenta NK comparisons, genes in liver NK cells had relatively reduced significance and log2FC. We also observed upregulation of AUTS2 and TXK in liver NK cells, both of which been associated with poor prognosis and malignant progression in multiple human cancers (36, 37). Additional differentiation-related genes were confirmed in placenta NK cells, such as IL1R1, which has been shown to play a role in precursor commitment to the NK cell lineage (38). These data are consistent with tissue-specific gene signatures for canine NK cells despite moderate functional overlap. In addition, these data underscore the plasticity of canine NK cells across tissues and organs related to maturation and activation states.

### Canine tissue and peripheral NK cells can be categorized into distinct subsets

**Figure 3:**
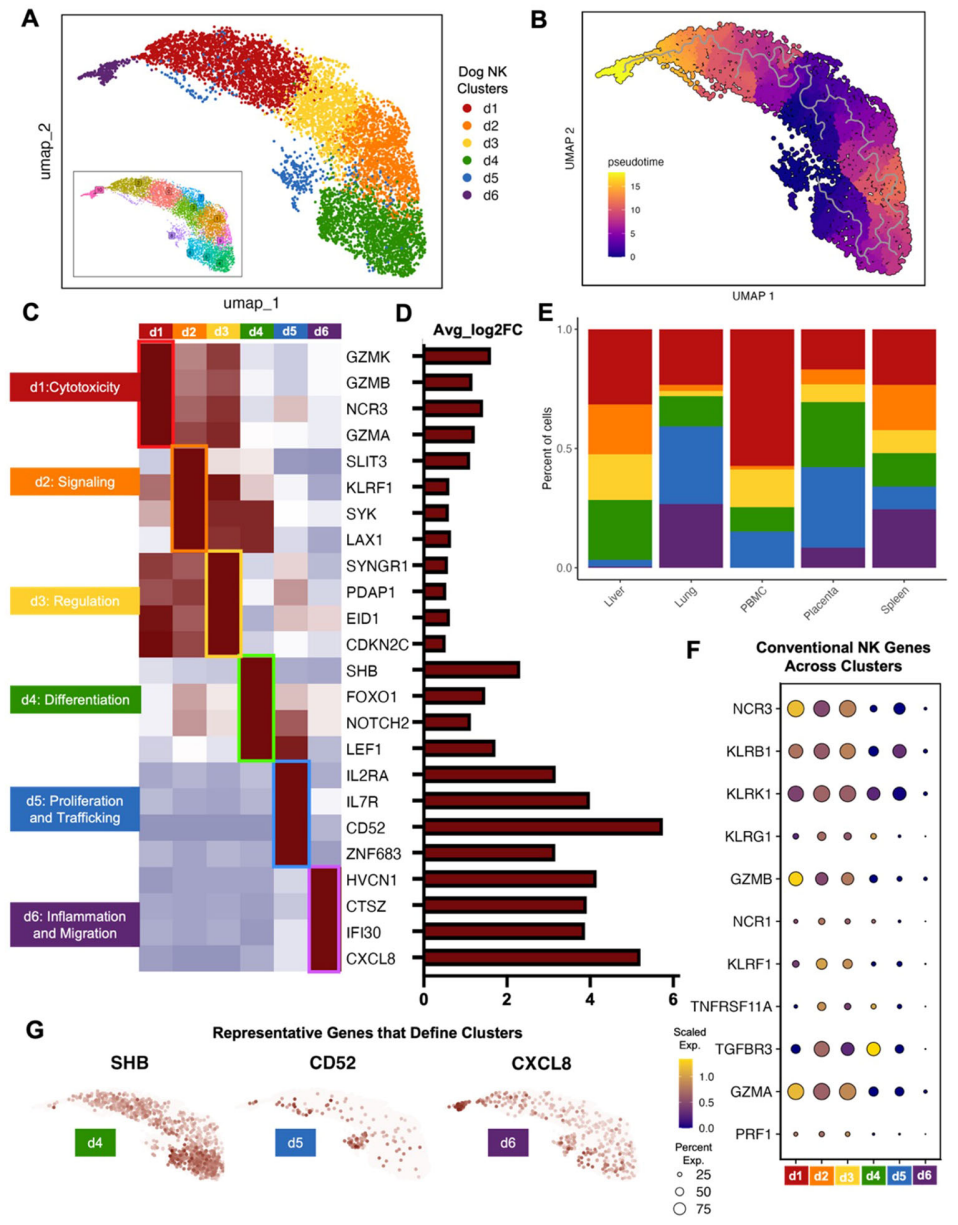
Canine tissue and peripheral NK cells can be categorized into distinct subsets. **(A)** Clusters resulting from unsupervised clustering were combined based on overlapping genes markers. The result was six canine NK cell clusters labeled d1-d6. **(B)** Cell trajectories projected onto the UMAP and colored by pseudotime. **(C, D)** Significant genes that distinguish each canine NK subcluster from the remaining subclusters visualized by **(C)** average expression heatmap and **(D)** average log2fold change bar plot. **(E)** Bar plots depicting the percent of cells grouped by canine NK subcluster and split by tissue compartment. **(F)** Dotplot of conventional NK cell markers and their expression within cells of each canine NK cell subcluster. **(G)** Feature plots depicting expression and distribution of selected significant genes that differentiate clusters d4 (left), d5 (center), and d6 (right).

Given the known diversity of human NK cells across peripheral blood and tissue, we then evaluated the heterogeneity of canine NK cell subsets to determine if similar heterogeneity to human NK cells was present. Canine NK clusters with overlapping genes were combined into 6 dog NK clusters labeled d1-6 (**Figure 3A**). Using pseudotime analysis, we determined that clusters d1 and d6 were furthest along the inferred trajectory (**Figure 3B**). The pseudotime trajectory could then be contextualized by classification of subclusters by genes that were best able to distinguish one cluster from another. These classifications included cytotoxicity, signaling, regulation, differentiation, proliferation and trafficking, and inflammation and migration (**Figures 3C, D**). The determination of a mature, cytotoxic subset, d1, showed high alignment with the CD56^dim^CD16^bright^ subset of human NK cells based on their effector functions and predominance in human peripheral blood (**Figure 3E**). Generally, genes defining canine clusters d1, d2, and d3 were expressed in multiple clusters with high expression of conventional NK genes, while genes in d4, d5, and d6 had unique gene expression with higher fold change in gene expression compared to the other clusters (**Figures 3D, F, G**). In particular, certain NK cell clusters had lower relative expression of classic NK cell markers compared to other NK cells, such as cluster d6, but still expressed NK markers significantly higher than the non-NK populations. The cells that made up NK cluster d6 were highly enriched for genes known to be involved in inflammatory processes compared to the remaining NK cell clusters. All subclusters were present across tissues except for d6, which clustered farthest along the pseudotime trajectory, was absent in PBMCs, and showed only minimal presence in liver NK cells. Notably, the d6 cluster was most abundant in lung NK cells, consistent with genes associated with inflammatory response and infiltration, including CXCL8 which was one of the top five significant genes in lung NK cells, suggesting a highly specialized function for this NK cluster in lung mucosal immunology. Genes involved in proliferation and trafficking were representative of cluster d5, and these clusters were most abundant in lung and placenta NK cells. Notable differential gene expression in this cluster included CD52 and IL7R, markers of CD56^bright^ NK cells in humans (10, 31, 39). As expected, the cluster associated with differentiation, d4, was largely found in the placenta as well as in liver NK cells. Genes in cluster d4 have also been associated with CD56^bright^ features in humans, such as LEF1, but also included genes specific to stem cells, such as NOTCH2 found in human decidual NK cells (40). Overall, the dog NK clusters demonstrated significant heterogeneity consistent with human NK heterogeneity (28, 41–43), consistent with gene signatures that aligned with our distinct canine tissue-specific NK signatures and also overlapped with established NK subtypes in humans.

### Human NK cells similar to dog NK cells in abundance and immune interactions across tissues

**Figure 4:**
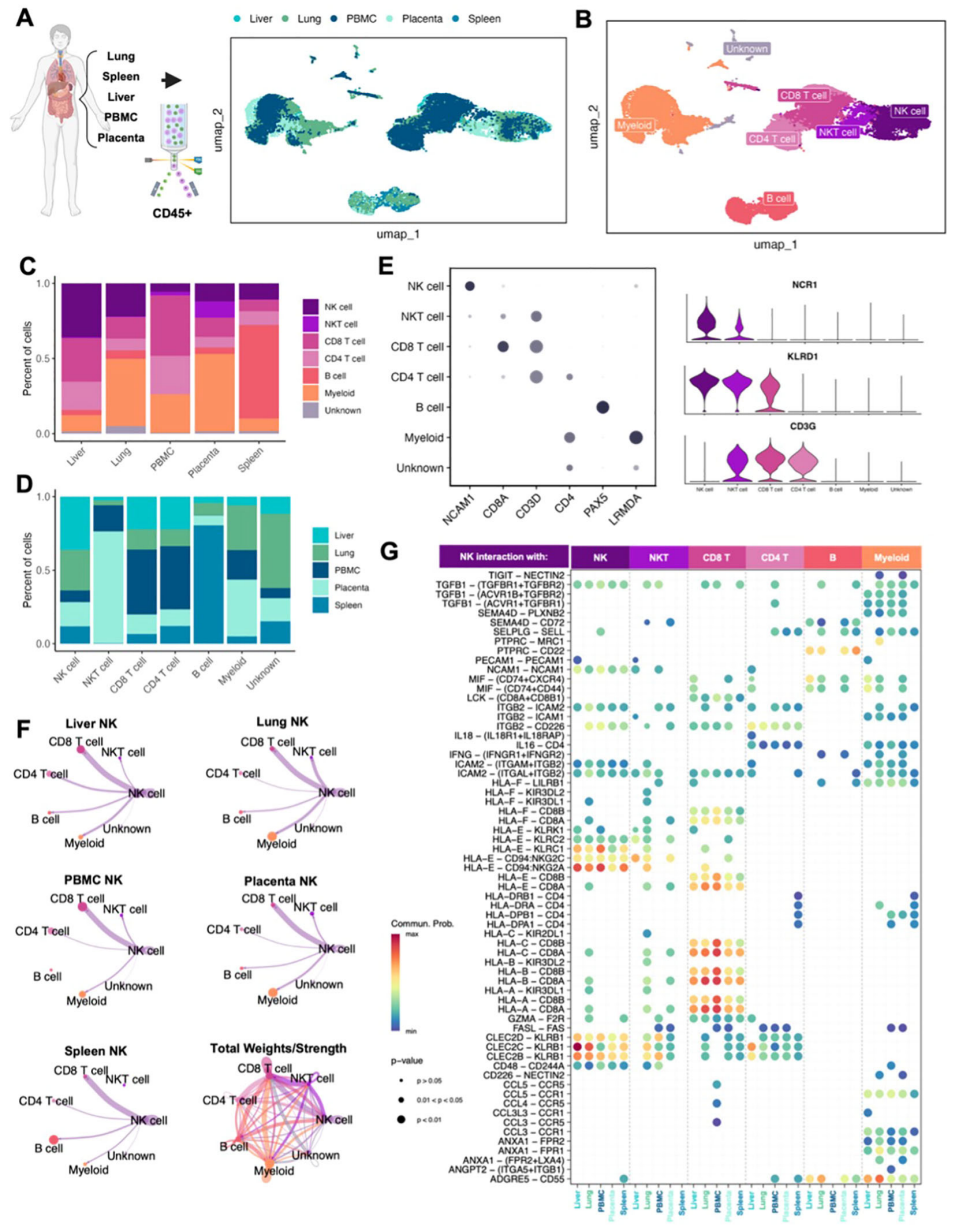
Human NK cells similar to dog NK cells in abundance and immune interactions across tissues. **(A, B)** Schema depicting the human samples obtained and the sorting and sequencing of CD45+ immune cells. Integration of five total samples were visualized by UMAP and color coded by **(A)** tissue or **(B)** cell type. **(C, D)** Bar plots depicting the percent of cells split by **(C)** tissue compartment or **(D)** cell type. **(E)** Left: dotplot of representative canonical gene markers used to confirm cell types. Dot color represents average gene expression and dot size represents the percent of cells expressing the gene. Right: violin plot of additional genes distinguishing NK cells from T lymphocytes. **(F)** Circle plots constructed using CellChat visualizing predicted NK cell outgoing interactions separated by tissue. Lines represent the scaled weights or strength of the interaction. Total weights and strengths of ingoing and outgoing interactions between all cell types across all tissues visualized in the bottom right panel. **(G)** Bubbleplot of significant predicted ligand receptor interactions between NK cells and other cell types, separated by tissue. Color of dot represents the probability of the interaction; size of the dot represents p-value. Only interactions expressed in a minimum of 10 cells were retained in the analysis.

The insights gained from the characterization of canine NK cells can improve the comparative model and help advance NK immunotherapy across species. To validate the results of our canine NK data, we created an equivalent transcriptomic atlas of human NK cells across tissues and peripheral blood. We acquired samples of placenta, spleen, liver, lung, and blood from healthy/non-cancer bearing human patients (**Supplementary Table 2**). The tissues were dissociated into single cell suspension and sorted for CD45+ immune cells before submitting for scRNAseq. After stringent quality control, processing, and integration to remove batch effects, approximately 34,000 cells were available for analysis and visualization by UMAP (**Figures 4A, B**). Expected cell types were found across the tissues, including a NKT cell cluster that was not apparent in our canine analysis. This NKT cluster was particularly evident in human placenta where it is thought to play a crucial role in immune surveillance during pregnancy (44) (**Figures 4B, C**). We identified 5,491 human NK cells across our tissue samples, the majority again being from liver (n=1,985) but with significant contributions from the lung (n=1,514), followed by PBMC, placenta, and spleen with an average of 664 cells each. Similar to our canine samples, the largest human NK proportion was found in the liver, making up 36% of CD45+ cells, compared to the 44% found in canine liver. Although the absolute proportions of NK cells in other tissues were generally low across the analyzed human tissues similar to dogs, there were differences in NK proportions. (**Figures 4C, D**). Most notably, the lung had the second highest percent of NK cells in humans but the lowest percent of NK cells in the dog. This is particularly important given the unique role of lung-resident NK cells and the unique expression signatures identified in human lung NK cells that enable their functional role across pathologies and impact outcomes (27). The identity of human cell types was confirmed by known canonical markers, with the advantage of human NK cells having unique expression of CD56 (NCAM1) not expressed in canine NK cells (**Figure 4E**). We additionally found that NCR1 was a more reliable marker of human NK cells than canine NK cells, which had distinctly higher expression of NCR3. Once cell identities were annotated, we again used CellChat to interrogate the inferred interactions between cell types across tissues, highlighting distinct interactions with NK cells (**Figure 4F**). Intriguingly, unlike canine NK cells, human NK cells appeared to have particularly strong interactions with CD8 T cells across organs, likely representative of their coordinated roles in immune surveillance and anti-tumor/anti-viral responses (45, 46), with additional notable interactions between NK cells and both myeloid and NKT cells in the lung. Canine NK cells did not show a strong relationship with canine CD8 T cells, but we did notice stronger interactions with myeloid cells in the lung compared to human. The total interactions depict a similarly complex network of cell interactions in both species with important differences in certain cell-type communications, most notably with myeloid and CD8 T cells. We then queried the ligand-receptor interactions that were significant within the cell communication network to discern the states of the NK cells based on their outgoing signals. There were greater numbers of significant NK receptor-ligand interactions in human than in canine tissues with 65 and 29, respectively (**Figure 4G**). However, certain conserved patterns emerged across species, including the interaction of PTPRC-MRC1 between myeloid and NK cells in the lung, which was highly frequent in both human and dog. We also observed high probability of interaction of CLEC2D-KLRB1 between NK cells and CD8 T cells across all tissues in both human and dog. The multiple HLA-dependent interactions between NK cells and CD8 T cells were particularly striking in our human data, with the greatest probability occurring between classical HLA class I molecules on NK cells and CD8A/B on CD8 T cells in the peripheral blood. While there was considerable overlap in receptor interactions between NK cells in dogs and humans, we also observed key differences. For example, TIGIT-NECTIN2 was a significant interaction between NK and myeloid cells in humans which we did not observe in canine NK cells. TIGIT is a critical immune checkpoint receptor in humans which we and others have identified, with potential for immunotherapy targeting (18, 47). The significance of this receptor in canine NK cells requires further investigation and may have implications for comparative work. Overall, our results suggest notable cross-species similarities in NK proportions and cell interactions and further underline the homology of NK immunogenomics between dogs and humans, especially when compared to murine counterparts (9).

## Discussion

In this study, we demonstrate varying abundance and genomic profiles of canine NK cells across diverse tissues including blood, spleen, liver, lung, placenta. We identified distinct canine NK subpopulations throughout tissue compartments with variations in gene expression associated with cytotoxicity, signaling, regulation, and maturation, consistent with gene programs adapted to their tissue of residence. NK cells in the placenta upregulated markers associated with differentiation, NK cells in the lung upregulated activation and migration markers, and NK cells in the liver had a mixed signature with specific regulatory genes. Importantly, we also observed large populations of NK cells in the liver and strong interactions between NK cells and myeloid cells in the lung, especially for dogs. NK cell abundances and cell interactions were compared to analogous human samples, and we observed notable similarities and illuminating differences. Notably, there were reproducibly strong interactions between NK cells and CD8+ cells across human tissues which less distinct in dogs, but interactions such as PTPRC-MRC1 were conserved across species. Together, we present the first in-depth transcriptomic analysis of tissue-resident canine NK cells, elucidating their diversity and malleability, role as key immune constituents of canine tissues, and validity as a model of human NK cells. The heterogeneity of human NK cells has been well-documented in both peripheral blood and tissue (13, 25, 28, 32), and we observed similar variability in canine NK cells across blood and tissues, although within individual organs there were reproducible findings among individual NK cells consistent with tissue resident phenotypes. We found distinct signatures that represent NK cells across tissues, especially in the lung, placenta, and liver. Additionally, we identified six canine NK cell subsets that were present across tissues, implying the tunability of NK cells based on the tissue environment and cell signals present (46, 48). NK cells in the lung and placenta demonstrated activated/cytotoxic and undifferentiated gene signatures, respectively, whereas in the canine liver, NK cells demonstrated features of both activation and immunoregulation.

This detailed immunogenomic evaluation of canine NK subsets is not only impactful in better characterizing the comparative scope of canine models in immuno-oncology but also has direct translational relevance for advancing adoptive NK therapy in both dogs and people. Donor sources and genetic modifications of human NK cells for adoptive cell therapy have continued to advance in recent years (49–54), and numerous methods are being employed to enhance antitumor efficacy through mechanisms including blocking inhibitory receptors with monoclonal antibodies, amplifying activation with bi- and tri-specific killer engagers (BiKEs and TriKEs), and increasing persistence with cytokine support. NK cell sources for cellular therapy include umbilical cord blood (CB), induced pluripotent stem cells (iPSCs), as well as peripheral blood and cell lines such as NK-92 (53, 55, 56). Cord blood-derived human NK cells are thought to have higher proliferative capabilities than those in the peripheral blood and, along with immature iPSC-derived NK cells, are more readily engineered to create scalable NK cell products, including chimeric antigen receptor (CAR) NK cells. Following this line of reasoning, we characterize canine NK cells derived from the placenta as expressing markers corresponding with early development while maintaining indicators of signaling and activation. Therefore, canine placental NK cells with undifferentiated properties could be promising candidates for novel immunotherapy approaches, particularly given recent studies showing successful adoptive transfer of allogeneic PBMC-derived canine NK cells with acceptable side effects (17). A clearer understanding of canine NK cell biology will lay the foundation for improved design and implementation of canine NK cell therapy.

Accurate models of human NK cells and related immunotherapies are crucial for the forward momentum of the field, especially given the limitations of murine models (57, 58). While companion dogs are increasingly viewed as innovative models of human cancers, only recently have canine NK cells been thoroughly studied though limited to NK cells in the blood (9). Canine NK cells are notoriously difficult to identify, mostly due to their lack of reproducible cell surface marker expression such as CD56 in humans or NK1.1 in C57/BL6 mice. There has been moderate success identifying canine NK cells by NKp46, the pan-mammalian NK cell marker, using flow cytometry. NKp46 is a transmembrane receptor encoded by the gene NCR1, part of a family of natural cytotoxicity receptors (NCRs) that include NKp44 and NKp30, encoded by NCR2 and NCR3, respectively. Interestingly, we and others have found that in transcriptomic analysis, NCR3 is more highly expressed in canine NK cells than NCR1. Our results are similar to those of Ammons et al. who observed from circulating leukocytes in healthy and cancer bearing dogs that NCR3, rather than NCR1, was more indicative of the transcriptional signature of canine NK cells (21). In contrast to NCR1, NCR3 is not constitutively expressed by NK cells in all mammals and has been identified as a pseudogene in most mouse strains (59). Additionally, while NCR1 is always activating, NCR3 can be activating or inhibitory based on the splice variants NKp30A, NKp30B or NKp30C (60, 61), although several studies associate NCR3 primarily with increased activation. Human NK cells express both NCR1 and NCR3 at high levels (28), and low expression of NCR3 specifically has been associated with poor prognosis in lung cancer (61). The expression of NCR3 as a canonical marker of canine NK cells represents an important example of the value of comparative studies between human and canine NK cells, highlighting the need to understand both the similarities and differences between human and canine NK biology. In this comparative analysis it is crucial to acknowledge the nuances of bioinformatic comparisons between human and canine NK cells, especially given the different markers used to identify NK cells in each species. Ultimately, additional work is needed to understand the immunological and clinical significance of these similarities and differences, particularly in regard to scRNAseq analyses. These data represent a basis through which further analyses of canine models of human diseases can be explored, especially in relation to treatment and clinical trials.

Overall, our single cell atlas of circulation and tissue-resident canine NK cells reveals the variety of canine NK cell states and how they are tailored to their specific tissue resident environment. Our comprehensive transcriptomic analysis provides novel insights into canine NK profiles across tissues with important comparison to their human counterparts. This investigation increases our understanding of both human and canine NK cells, advances comparative NK cell biology and the canine model, and lays the groundwork for future exploration of NK cell sources and biomarkers of response for improved immunotherapy.

## Data Availability

The data presented in the study are deposited in the National Center for Biotechnology Information Sequence Read Archive repository, accession number PRJNA1259073.
