# Supplementary material for: Single cell atlas of canine natural killer cells identifies distinct circulating and tissue resident gene profiles
Source: Front Immunol. 2025 May 15;16:1571085. doi: 10.3389/fimmu.2025.1571085 (PMC12119461; doi:10.3389/fimmu.2025.1571085)
Supplement: Supplementary file 1 [file DataSheet1.pdf]

SUPPLEMENTAL FIGURES

Supplemental Figure 1: Characteristics of canine sample donors.

Sample	Breed	Sex	Age	Diagnosis	Procedure
Lung	Beagle	FS	10	Pulmonary Adenoma	Lung Lobectomy
Lung	Australian Shepherd	FS	10	Pulmonary Adenocarcinoma	Lung Lobectomy
Liver	Pit Bull Terrier	FS	9	Poorly Differentiated Sarcoma	Liver Lobectomy
Liver	Pomeranian Mix	MN	10	Hematocellular Adenoma	Liver Lobectomy
Spleen	Bull Mastiff	FS	9	Lymphoma	Splenectomy
Spleen	Border Collie	FS	14	Hematoma	Splenectomy
Placenta	Danish-Swedish Farmdog	F	5	Pregnancy	C-section
Placenta	French Bulldog	F	4	Pregnancy	C-section
PBMC	Beagle	FS	6	Healthy	None

Supplemental Figure 2: Characteristics of human sample donors.

Sample	Sex	Age	Diagnosis	Procedure
Lung	M	65	Stage IA Lung Adenocarcinoma	Lung Lobectomy
Liver	M	42	Benign Hemangioma	Partial Hepatectomy
Spleen	M	72	Stage IIB Pancreatic Carcinoma	Splenectomy
Placenta	F	32	Pregnancy	Vaginal Delivery
PBMC	M	58	Rhabdomyosarcoma	Sarcoma Resection
